# Purification and biochemical properties of a thermostable, haloalkaline cellulase from *Bacillus licheniformis *AMF-07 and its application for hydrolysis of different cellulosic substrates to bioethanol production

**Published:** 2016-09

**Authors:** Fatemeh Azadian, Arastoo Badoei-dalfard, Abdolhamid Namaki-Shoushtari, Mehdi Hassanshahian

**Affiliations:** Department of Biology, Faculty of Sciences, Shahid Bahonar University of Kerman, Kerman, Iran

**Keywords:** Cellulosic hydrolysate, Bioethanol, Purification, Characterization

## Abstract

A thermophilic strain AMF-07, hydrolyzing carboxymethylcellulose (CMC) was isolated from Kerman hot spring and was identified as *Bacillus licheniformis *based on 16S rRNA sequence homology. The carboxymethylcellulase (CMCase) enzyme produced by the *B. licheniformis *was purified by (NH4)2SO4 precipitation, ion exchange and gel filtration chromatography. The purified enzyme gave a single band on SDS- PAGE with a molecular weight of 37 kDa. The CMCase enzyme was highly active and stable over broad ranges of temperature (40-80ºC), pH (6.0-10.0) and NaCl concentration (10-25%) with an optimum at 70ºC, pH 9.0 and 20% NaCl, which showed excellent thermostable, alkali-stable and halostable properties. Moreover, it displayed high activity in the presence of cyclohexane (134%) and chloroform (120%). Saccharification of rice bran and wheat bran by the CMCase enzyme resulted in respective yields of 24 and 32 g L-1 reducing sugars. The enzymatic hydrolysates of rice bran were then used as the substrate for ethanol production by *Saccharomyces cerevisiae*. Fermentation of cellulosic hydrolysate using *S. cerevisiae*, reached maximum ethanol production about 0.125 g g-1 dry substrate (pretreated wheat bran). Thus, the purified cellulase from *B. licheniformis *AMF-07 utilizing lignocellulosic biomass could be greatly useful to develop industrial processes.

## INTRODUCTION

Cellulose is the most abundant and renewable organic material that can be converted to glucose for bioethanol production [1]. Developing processes for effectively converting agricultural wastes for production of high value chemicals has gained considerable interest [2, 3]. Cellulases are important industrial enzymes for the most bioconversion processes [4]. Cellulases are broadly utilized in various industrial processes such as food, brewery, wine, pulp and paper, textile, detergent, feed and agriculture [5]. Bioethanol production from lignocellulosic material, such as green feedstock, is expected to be an alternative energy source in the future [6]. A cellulosic enzyme system contains at least three major types of enzymes, including endo [1, 4] beta- D glucanase (EC 3.2.1.4), exo (1, 4) beta-D glucanase (EC 3.2.1.91) and beta-glucosidase (EC 3.2.1.21) [7]. Bacterial cellulases could provide a key opportunity for application in various industries [8]. Several *Bacillus *strains have been recently reported for cellulytic activities including *B. sphaericus *JS1 [9], *Bacillus carboniphilus, *CAS *3 *[10], *Bacillus *sp. L1 [11], *B. halodurans *CAS 1 [12]. Bacteria inhabit a wide variety of environments and have received more attention as sources for novel cellulases. Enzymes that can function at extreme environmental conditions including pH, temperature, salts and various organic solvents have major biotechnological advantages [13]. Thermophilic enzymes are usually optimally active between 60 and 80 ◦C. With their great stability and activities at high temperatures, thermophilic enzymes have major biotechnological advantages over mesophilic enzymes [14]. This study involves the purification and characterization of a cellulase from *B. licheniformis *strain AMF-07, which was isolated from Kerman hot spring. In addition, its application in ethanol production was evaluated by fermenting enzymatic hydrolysates using *Saccharomyces cerevisiae.*

## MATERIALS AND METHODS

**Isolation and identification of strain AMF-07: **Water samples were collected from different sites of hot spring for the isolation of cellulose degrading bacteria. A CMC medium that contained K2HPO4 0.1%, KCl 1%, NaNO3 0.1% , yeast exract 0.05%, MgSO4.7H2O 0.05%, glucose 0.1% supplemented with 0.5% (w/v) carboxymethyl- cellulose (CMC), was used. The pH of the agar plate was adjusted to 7.5 using 1M NaOH. After incubation, the plates were stained by 1% (v/w) Congo red solution for 15–20 min, then washed with a 1 M NaCl solution for 15–20 min [15]. The isolate that had a clear hydrolysis zone on the CMC medium plate was chosen in subsequent studies and designated AMF-07 [16]. Genomic DNA for molecular identification of bacterial strain was extracted by phenol-chloroform method [17]. PCR was conducted using two universal primers for the bacterial 16S rRNA gene. The PCR amplification of 16S rRNA was carried out in 25 µl reaction mixture containing H2O, 10X buffer (1X), dNTP (200 µM), MgCl2 (0.2 mM), Taq DNA polymerase (0.05 units/µL), template DNA (200 pg/µL) and 0.2 µM of universal forward primer (5-AGT TTG ATC CTG GCT CAG-3) and reverse primer (5-GGC ACC TTG TTA CGA CTT-3). The PCR products were separated by 1% agarose gel electrophoresis. Amplicons were later sequenced and compared with sequence in nucleotide database (NCBI) using the BLAST algorithm. The neighbor-joining phylogenetic analysis was carried out with MEGA4 program [18].

**Enzyme assay: **Celulase activity was measured by the 3, 5 dinitrosalicylic acid (DNS) method [19]. CMCase activity was determined by incubation 500 µl of 1% CMC in 50 mM sodium phosphate buffer (pH 7.5) with 500 µl cell free culture for 30 min at 50 ◦C. The reaction was stopped by adding the 1 ml 3, 5 dinitrosalicylic acid (DNS) reagent and boiled in a water bath for 10 min. After cooling at room temperature, the amount of glucose released was determined by measuring absorbance at 540 nm. CMCase activity was determined by using a calibration curve for glucose. One unit of enzyme activity was defined as the amount of enzyme that releases 1 µmol reducing sugars per min.

**Production and purification of CMCase: **For CMCase production, *Bacillus licheniformis *AMF-07 was grown in an Erlenmeyer flask (500 ml) containing 150 ml of optimum medium supplemented with 0.5% CMC (pH 6.0) was seeded with 5% 16 h old pre-culture and incubated (160 rpm) for 72 h at 60 ◦C. After incubation, culture broth was centrifuged (4ºC and 10,000 × g for 15 min), and the supernatant was used as crude enzyme for further purification. The supernatant was precipitated with ammonium sulfate (85%) at 4ºC. The precipitates were collected through centrifugation at 12000 g for 10 min. The pellets were dissolved in a minimum volume of 50 mM sodium phosphate buffer (pH 7.5) and were dialyzed against the same buffer at 4ºC. The dialysate enzyme was subjected to Q-Sepharose column chromatography (5 cm × 20 cm) which equilibrated with 50 mM sodium phosphate buffer (pH 7.5) at flow rate 0.5 ml/min. The bound proteins were eluted with a linear gradient of NaCl (0.1-0.5 M) in the equilibration buffer. Fractions which displayed cellulase activity were pooled together and concentrated by ammonium sulfate precipitation with the same procedure. The resulting precipitate was collected by centrifugation and dissolved in 50 mM Tris–HCl buffer (pH 7.5). Concentrated fractions were loaded onto a Sephadex G-100 column (2.5 cm × 50 cm) equilibrated with 50 mM Tris–HCl buffer (pH 7.5) and eluted with the same buffer at a flow rate of 0.2 ml/min. Fractions exhibiting CMCase activity were pooled and used as a purified enzyme for the following studies.

**SDS-PAGE and zymogram analysis: **Sodium dodecyl sulfate-polyacrylamide gel electrophoresis (SDS-PAGE) was performed with 10% polyacrylamide gel by the method of Laemmli with some modification [20]. Gels were visualized by silver staining protocol of Hames and Rickwood [21]. The CMCase zymogram analyses were performed by using a 0.1% CMC (w/v) incorporated into the polyacrylamide. The CMC was mixed with polyacrylamide during gel preparation. Following SDS-PAGE, the gels were washed four times at 4 ºC for 30 min in renaturation buffer (20 mM KH2PO4/NaOH, pH 8.0). The first two washes contained 25% (v/v) isopropanol. The clearing zones that corresponded to enzyme activities were visualized using 0.5% (w/v) Congo red.

**Influence of pH, temperature and salinity on CMCase activity and stability: **The optimal temperature of the purified enzyme was examined in 50 mM sodium phosphate buffer (pH 7.5) under different temperature between 30 and 70ºC. At each temperature, the purified enzyme was incubated with 500 µl of the CMC (1%) for 30 min. For thermal stability, the purified enzyme (0.5 ml) was incubated at temperatures of 30-70ºC for 60 min. The residual enzyme activity was determined as described above. The optimal pH of the purified enzyme for hydrolysis of CMC was examined by incubating the mixture of the purified enzyme and 1% (w/v) CMC dissolved in an appropriate buffer with different pHs: 50 mM of sodium acetate (pH 3.0-6.0), sodium phosphate (pH 7.0-8.0), Tris-base (9.0-10.0) and glycine-NaOH (pH 11.0-12.0). The pH stability was determined by pre-incubating the purified enzyme in different buffer systems at 60ºC for 60 min, and residual activity was determined under optimal assay conditions. The optimum NaCl concentration for CMCase activity was determined by incubating the purified enzyme with substrate solution (1% CMC) for 1 h at different NaCl concentrations (0–30%). The halo-stability of purified enzyme was examined by incubating the purified enzyme in 50 mM sodium phosphate buffer (pH 7.5) at various NaCl concentrations (0–30%) for 60 min and the residual activity were assayed by standard DNS method [19].

**Activity of the enzyme on different cellulosic substrates: **The agriculture wastes such as corn stover (C.S), wheat straw (W.S), rice bran (R.B), alfalfa straw (A.S), wheat bran (W.B), wheat straw:corn stover (W.S:C.S) and corn stover: alfalfa straw (C.S:A.S) were firstly dried and cut into small pieces and then they were pretreated with NaOH (0.1 M) for 1 h at 120ºC in an autoclave. After cooling, the samples were washed several times in tap water and finally rinse using distilled water. Then, they were air-dried by spreading on paper. These pretreated biomasses were used for enzymatic hydrolysis [40, 41]. The reducing sugar liberated in the reaction mixture was measured by DNS method at 540 nm [19]. One unit of cellulase activity was defined as the amount of enzyme capable of releasing 1 µmol of glucose per minute under the assay condition.

**CMCase activity and stability in organic solvents and effect of various additive and commercial detergents on enzyme activity: **For solvent stability, purified enzyme was pre-incubated for 4h in different solvents (25%, v/v) such as methanol, chloroform, toluene, DMF, diethyl ether, n-butanol, DMSO and cyclohexane. Thereafter, residual activity was determined under optimized assay conditions considering control as 100%. The activity of purified enzyme was determined by incubating the purified enzyme with substrate solution (1% CMC) for 60 min at different organic solvents. The effect of various metal ions and reagents were examined on the activity of purified enzyme. The additives used in this study were the salts of K+ (KCl), Mn2+ (MnSO4), Fe2+ (FeSO4), Mg2+ (MgSO4), Cu2+ (CuSO4), Ca2+(CaCl2), Hg2+ (Hg2Cl2), Zn2+ (ZnSO4), Co2+ (CoCl2) and chemical reagents, ethylenediaminetetraacetic acid (EDTA), B-mercaptoethanol, SDS, Triton X-100, Tween 20, H2O2 and residual activity was calculated as relative (%) considering control as 100%. The effect of various local detergents on the CMCase activity was determined by the presence of Dioxygene, Shooma, Banoo, Barf, Darya, Kaf and Taj. Each local detergent was prepared in the in 50 mM sodium phosphate buffer (pH 7.5) and activity was measured as standard assay. Relative activity is expressed as a percentage of the activity level in the absence of detergents.

**Biomass saccharification and ethanol fermentation: **For preparation of feedstock material, the substrate was dried in oven at 70ºC to remove residual moisture. Lignocellulosic material were cut into small pieces. The samples were reacted with 0.1 M NaOH for 1 h at 120ºC in an autoclave. After cooling, this material was washed with water until to neutral pH. After washing, the samples were dried at 60°C for 72 h. The pretreated feed stock was used for hydrolysis experiments. Enzymatic saccharification of alkali pretreated W.B and R.B were performed in 150 ml stoppered conical flasks by incubating 2 g of biomass in 45 mL of sodium phosphate buffer (pH 7.0) with 20% NaCl, 1mL NaN3 (1%, w/v) and 2 mL purified enzyme solution. The samples were incubated at 70 ºC for 48 h in a shaking water bath. The fermentation was carried out at 30 ºC in 250 ml Erlenmeyer flask containing hydrolyzed W.B with the supplementation of 10% v/v of a 12 h old seed culture of *Saccharomyces cerevisiae*. The hydrolysate was used for reducing sugar analysis by 3, 5-dinitrosalicylic acid method [19]. Samples were collected at regular time points and centrifuged for 10 min at 4ºC at 10000 rpm. Bioethanol production was examined by Jones reagent (One milliliter of K2Cr2O7 (2 %), 5 ml of H2SO4) in the presence of 3 ml samples [22]. Ethanol was oxidized into acetic acid with potassium dichromate in the presence of sulfuric acid and gave blue-green color which indicates positive test [23].

## RESULTS AND DISCUSSION

**Figure 1:**
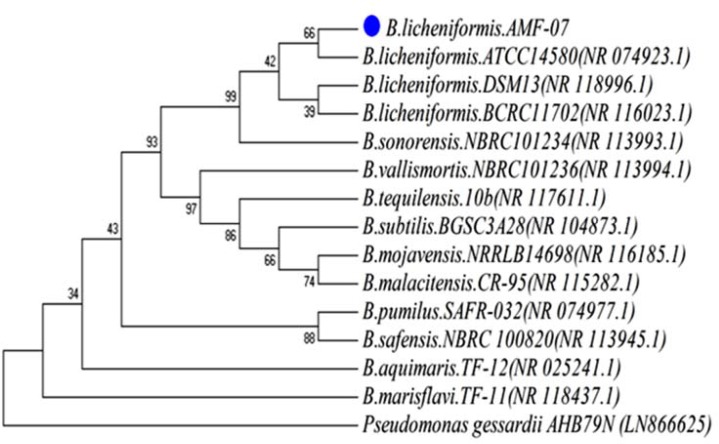
The phylogenetic tree for *Bacillus licheniformis *AMF-07 and related strains based on the 16S rRNA sequence. The tree was drawn using MEGA 4.0 with computing linearized tree

*Bacillus licheniformis *AMF-07, isolated from Gorooh hot spring exhibited evident clear zones around the colonies on CMC agar plates following staining with 1% Congo red solution, indicating that it secretes prominent amounts of CMCase. The biochemical characterization of the thermostable AMF-07 strain revealed the organism as gram- positive, aerobic and motile bacteria. The 16S rRNA gene sequencing analysis evidenced that this strain exhibited highest homology (99%) with *Bacillus licheniformis *strain AMF-07 (Fig. 1).

**Figure 2:**
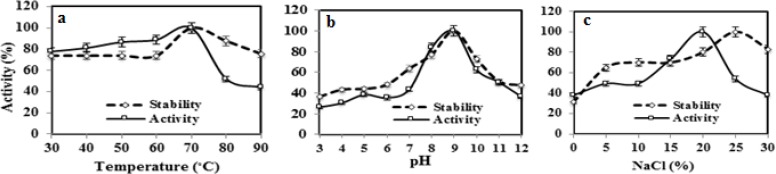
Effect of (a) temperature, (b) pH and (c) NaCl on the activity and stability of the purified CMCase AMF-07. The activity of purified CMCase was measured at different temperatures, pH and NaCl concentrations using a phosphate buffer (pH 7.5). Relative activity was defined as the percentage of activity detected with respect to the maximum enzyme activity. For determining the stability, the activity of the enzyme without any treatment was taken as 100%.

Based on the evolution distance and the phylogenetic tree, this strain was identified as *Bacillus licheniformis *and designated as *Bacillus licheniformis *AMF-07. Its nucleotide sequence deposited in Genbank as KX016597 accession number. In previous studies, diverse types of genera have been reported for producing the cellulase enzyme including* B. subtilis *[24], *Marinobacter *[25], *Penicillium *[26]. It is mentioned that, *B. licheniformis *WBS1 (GU590781) and *Bacillus *sp. WBS3 were previously isolated from an Indian hot spring. These two isolates produced cellulase when cultivated at 55°C in liquid culture containing 3% carboxymethylcellulose [27]. Acharya et al., also reported that, the maximum cellulases production by both of these isolates was detected after 60 h incubation period using wheat and rice straw [28]. CMCase enzyme produced by the culture when grown on medium supplemented with 0.5% (w/v) CMC. The CMCase from the culture broth of *Bacillus licheniformis *AMF-07 was purified through multistep purification, ammonium sulfate precipitation, Q-Sepharose and gel filtration chromatography. The overall purification fold of the enzyme was about 8.85 with the specific activity of 412.32 U/mg. The purity was confirmed by SDS-PAGE showing a single protein band of molecular weight about 37 kDa which is smaller than cellulases from other *Bacillus *strains such as *Bacillus subtilis *subsp. subtilis A-53 (56 kDa) [23], *Bacillus circulans *(43 kDa) [26] and *Bacillus flexus *(97 kDa) [29], but larger than the cellulase from *Bacillus sphaericus *JS1 (29 kDa) [9]. The zymogram exhibited a single significant activity band of cellulase suggesting that the enzyme was able to hydrolyze the sodium carboxymethyl cellulose and had only one isoform. The activity profile of purified enzyme showed the optimum residual activity at pH 9.0, temperature 70ºC and NaCl concentration (20-30%). The effect of the temperature in the stability of enzymes is shown in Figure 2a.

The enzyme remained 100% stable up to 70°C, and started to undergo denaturation after that. The thermo-stability of the purified cellulase was similar to the cellulase of *B. halodurans *CAS 1 [12] and comparatively higher than cellulase produced by *B. subtilis *subsp. *subtilis *A-53 [23] and *B. licheniformis *AU 01 [30]. However, after treatment under temperature above 80ºC the enzyme activity was extremely reduced. Thermo-stable enzymes such as cellulases are advantageous for some applications, because higher processing temperatures can be employed, with consequent faster reaction rates, improved hydrolysis of cellulosic substrates, and reduced incidence of microbial contamination from mesophilic organisms [31]. Because of thermophilic nature of this enzyme, all CMCase activity measurement was determined at 50ºC. The enzyme was found to be stable in the broad range of pH (6.0–9.0). At higher alkaline pH enzyme retained 68% activity at incubation pH of 10.0 (Fig. 2b). These results maybe have shown the correlation between temperature and pH. The present results correlate well with earlier reports for alkaline extracellular cellulase reported from *B. flexus *[29], *B. sphaericus *JS1 [9], *Bacillus sp. *HSH-810 [32]. The halo stability studies revealed that the purified cellulase was highly stable even at 30% NaCl (Fig. 2c) which is similar to the cellulase produced by *B. halodurans *CAS1 [12]. Cellulose pretreatment lead to the extreme conditions such as high temperature, salinity or pH. The higher stability of the purified cellulase towards temperature (70ºC), pH (9.0) and NaCl (30%) could make this enzyme as an ideal candidate for enzymatic saccharification of cellulosic wastes to produce reducing sugars for further ethanol production. The relative hydrolytic activities of purified enzyme with different substrates were compared by measuring the amount of hydrolyzed products. The purified cellulase exhibited significantly higher activity towards CMC (100±0.8) followed by avicel (22±0.1) and cellobiose (15±0.7) (Table 1). Maximal activity was observed against CMC. These results suggest the nature of our enzyme as an endo type of cellulase. Similar finding was reported from *Bacillus *sp. L1 [11]. For the lignocellulosic wastes, corn stover (C.S), wheat straw (W.S), rice bran (R.B), alfalfa straw (A.S), wheat bran (W.B), wheat straw:corn stover (W.S:C.S) and corn stover:alfalfa straw (C.S:A.S) were examined. Results from this study revealed that W.B and R.B was found as suitable substrate for CMCase activity than other substrates (Table 1).

**Table 1:** Activity of CMCase on different cellulosic substrates

**Substrates**	**Activity (%)**	**Dry weight (mg.ml‾¹)**
CMC	100 ± 0.08	70
Avicel	22 ± 0.1	10
Cellobiose	15 ± 0.7	5
A.S	57 ± 0.9	76
C.S	74 ± 0.8	36
W.S	58 ± 1.2	44
R.B	100 ± 1.2	80
W.B	127 ± 1.1	90
C.S:A.S	76 ± 1.2	60
W.S:C.S	79 ± 0.99	42

Enzyme activity was measured in the presence of different substrates. Corn stover (C.S), wheat straw (W.S), rice bran (R.B), alfalfa straw (A.S) wheat bran (W.B), wheat straw: corn stover (W.S:C.S) and corn stover: alfalfa straw (C.S:A.S

Lee et al. (2010) found that rice bran was the best carbon source for cell growth and cellulase production by *Bacillus subtilis *subsp. subtlis A-5 [33]. Jo et al and Mayende et al., were also reported that rice hulls and rice bran were the best agricultural biomass for CMCase production by *B. amyloliquefaciens *DL-3 and *Bacillus *sp. CH 43, respectively [34, 35]. Hydrolysis of cellulosic biomass by the CMCase produced by *Bacillus licheniformis *AMF-07 could be useful for the production of ethanol, single cell protein and other industrially important chemicals. The organic solvent stable cellulases could be potentially useful for industrial processes such as bioremediation of chemically polluted salt marshes [36]. In view of that, stability of the purified CMCase of *Bacillus licheniformis *AMF-07 was studied and the results showed that no complete inactivation was observed in the presence of the organic solvents. The enzyme showed 34% increase in the activity in cyclohexane, 20% in chloroform followed DMSO (15%), toluene (13%), butanol (7%), and methanol (3%). However, a sharp decline in the residual activity was observed in DMF (51%) (Table 2).

**Table 2:** CMCase activity and stability in different organic solvents

**Organic solvents**	**Activity (%)**	**Stability (%)**
Control	100 ± 2.3	100 ± 1.8
Methanol	103 ± 1.6	96 ± 2.5
Chloroform	120 ± 1.6	146 ± 2.5
Toluene	113 ± 2.2	107 ± 1.8
DMF	49 ± 2.5	37 ± 2.5
Diethyl ether	100 ± 1.8	102 ± 2.5
Isopropanol	70 ± 2.3	94 ± 1.6
n-butanol	107 ± 1.7	87 ± 2.2
DMSO	115 ± 1.6	72 ± 1.8
Cyclohexane	134 ± 2.2	109 ± 2.2

The enzyme activity in the absence of organic solvents was taken as control (100%)

Cellulase from *B. aquimaris *showed 122 % activity in the presence of benzene [29]. Also the activity of enzyme Cel5A achieved from uncultured bacteria was also reported to be unaffected in presence of ethanol and methanol [37].The effect of various additives on the activity of purified CMCase was summarized in Table 3.

Presence of Ca+2 and Cu+2 metal ions in the reaction mixture stimulated the enzyme activity largely, while the metal ions of Mn+2, Zn+2 and Tx100 caused it to enhance moderately. The activity was found to be strongly inhibited by Co+2, Hg2+, K+, and H2O2. The activity was greatly inhibited by EDTA, indicated that the cellulase purified from this study was a metalloenzyme. Inhibition of the enzyme activity by Hg2+ was also reported from *B. sphaericus *JS1 [9], *Salinivibrio *NTU-05 [38]. The inhibition by Hg2+ ions is not just related to binding the thiol groups but may be the result of interactions with tryptophan residue or the carboxyl group of amino acids in the enzyme [39]. The purified CMCase from *B. licheniformis *AMF-07 retained its activity in the presence of some commercial detergents such as Dioxigene (122%) and Shooma (116%) and partially inhibited with Barf (90%), Kaf (85%), Taj (33%), and Darya (33%). (Table 4).

The purified CMCase from *B. halodurans *CAS1 retained its activity in the presence of some commercial detergents such as Rin (85.33%), Ariel (76.67%), Henko (64.67%) and Tide (80.33%) [19]. Among the 7 substrates tested, the purified cellulase was more active against wheat bran and rice bran than other biomasses (Table 1).

**Table 3:** Effect of metal ions and chemical reagents on CMCase activity

**Substances**	**Concentration (mM)**	**Residual activity (%)**
Control	-	100 ± 0.8
ZnSO4	5	103 ± 1.1
MgSO4	5	93 ± 0.8
FeSO4	5	98 ± 1.2
Hg2Cl2	5	30 ± 1.6
CaCl2	5	150 ± 1.7
CuSO4	5	160 ± 1.1
MnSO4	5	118 ± 1.6
KCl	5	40 ± 0.9
CoCl2	5	13 ± 0.8
SDS	5	60 ± 1.1
H2O2	5	10 ± 0.9
EDTA	5	0.5 ± 0.02
2Me	5	55 ± 0.7
Tx100	5	105 ± 1.2

Activity was measured in the presence of 5 mM of each compound as mention in material and methods. Enzyme activity in no metal ions considered as 100%.

**Table 4:** Activity of CMCase from *Bacillus* *licheniformis* AMF-07 with different commercial detergents

**Commercial detergents**	**Activity (%)**
Control	100± 0.7
Dioxigene	122± 0.9
Shooma	116± 1.2
Banoo	37± 0.8
Darya	33± 0.7
Barf	90± 1.1
Kaf	85± 0. 8
Taj	33± 0.9

**Figure 3:**
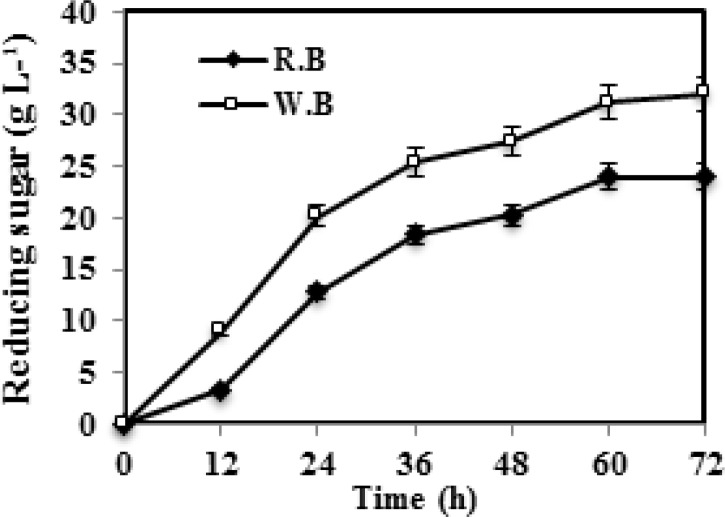
Time course profile of enzyme production by *Bacillus licheniformis *AMF-07 on wheat bran and rice bran. Results are the mean of three replicates

They were used for reducing sugar analysis. The samples were collected at different intervals and centrifuged at 10,000 rpm at 4°C for 10 min and the cell free supernatant was used for reducing sugar production. The amount of reducing sugar from wheat bran and rice bran were obtained as 32 g L-1 (72 h) and 24 g L-1 (60 h) (Fig. 3). Yu and Li studied enzymatic hydrolysis of corn stover and rice straw using cellulase from *Gracilibacillus *sp. SK1. The yield of reducing sugar from corn stover and rice straw were 27.1 g L-1(48 h) and 20.4 g L-1 (64 h) [40]. In this study, the total amounts of reducing sugars released from 1 g of dry feed stock were 0.8 g g-1 (dry substrate) for wheat bran and 0.6 g g-1 (dry substrate) for rice bran.. Liu et al. used the crude cellulases secreted by* fumigatus *Z5 for degradation of pretreated corn stover, and the yield of reducing sugar was 0.45 g g-1 (dry substrate) [41]. Yu and Li also reported, total amounts of reducing sugars released from 1 g of dry feed stock were 0.678 g g-1 (dry substrate) for corn stover and 0.502 g g-1 (dry substrate) for rice straw by using the crude enzyme from* Gracilibacillus *sp. SK1 [40].

**Figure 4:**
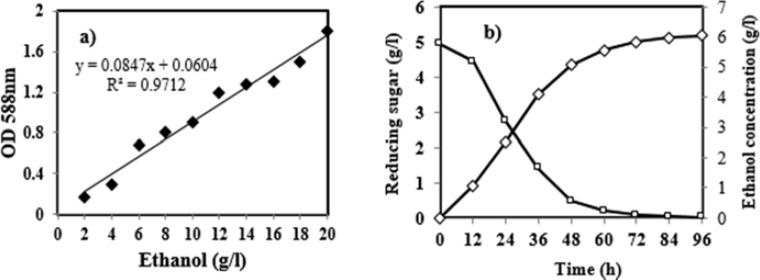
Calibration curve of ethanol standards (a), Changes in sugar concentration and ethanol contents of wheat bran pretreatment over the fermentation period by *Saccharomyces cerevisiae* (b

Time course of ethanol production using of the cellulase hydrolysate as the substrate was shown in Fig 4. Results show that, the ethanol production increased along with the fermentation period and maximal yield (5.17 g L-1) was observed at 60 h. In the fermentation using the hydrolysate, the glucose concentration reduced rapidly from 5.78 to 0.03 g L-1. This indicates that the amount of glucose produced decreased simultaneously by yeast cells was virtually in sync with the ethanol production. The yield of ethanol production by *B. licheniformis *AMF-07 was 0.125 g g-1 dry substrate (pretreated wheat bran). It is more than the yield of ethanol production by Sukumaran et al, which reported 0.093 g per gram dry substrate from pretreated rice straw using *Saccharomyces cerevisiae *[42]. Yu and Li also reported, the yield of ethanol was 0.186 g g-1 dry substrate (pretreated corn Stover) by using the crude enzyme from *Gracilibacillus *sp. SK1 [40]. These results suggested the prospects of the cellulase from* licheniformis *AMF-07 in application for bioethanol production.

In this study, *Bacillus licheniformis *AMF-07, capable of hydrolyzing CMC was isolated from Kerman hot spring. The in vitro functional activity was confirmed with zymogram and resulted molecular weight was about 37 kDa. The higher stability of the purified CMCase towards temperature (70ºC), pH (9.0) and NaCl (25%) could make this enzyme as a good candidate for enzymatic saccharification of cellulosic wastes to produce reducing sugars. The enzymatic hydrolysates of rice bran were then used as the substrate for ethanol production by *Saccharomyces cerevisiae*. The yield of ethanol production by* B. licheniformis *AMF-07 was 0.125 g g-1 dry substrate (pretreated wheat bran), which suggested the prospects of the cellulase from *B. licheniformis *AMF-07 in application for bioethanol production.
